# An assessment of human gastric fluid composition as a function of PPI usage

**DOI:** 10.14814/phy2.12269

**Published:** 2015-01-27

**Authors:** Emily Foltz, Sassan Azad, Mary Lou Everett, Zoie E. Holzknecht, Nathan L. Sanders, J. Will Thompson, Laura G. Dubois, William Parker, Shaf Keshavjee, Scott M. Palmer, R. Duane Davis, Shu S. Lin

**Affiliations:** Department of Surgery, Duke University Medical Center, Durham, North Carolina; Toronto General Research Institute, Toronto General Hospital, Toronto, Ontario, Canada; Department of Pharmacology and Cancer Biology, Duke University Medical Center, Durham, North Carolina; Duke Proteomics and Metabolomics Core Facility, Duke University Medical Center, Durham, North Carolina; Department of Medicine, Duke University Medical Center, Durham, North Carolina; Department of Immunology, Duke University Medical Center, Durham, North Carolina

**Keywords:** Bile, gastric fluid, gastricsin, proton pump inhibitors, trypsin

## Abstract

The standard of care for chronic gastro‐esophageal reflux disease (GERD), which affects up to 40% of the population, is the use of drugs such as proton pump inhibitors (PPIs) that block the production of stomach acid. Despite widespread use, the effects of PPIs on gastric fluid remain poorly characterized. In this study, gastric fluid was collected from patients undergoing cardiac surgery who were not (*n* = 40) or were (*n* = 25) actively taking PPIs. Various enzymatic and immunoassays as well as mass spectrometry were utilized to analyze the concentrations of bile, gastricsin, trypsin, and pepsin in the gastric fluid. Proteomic analyses by mass spectrometry suggested that degradation of trypsin at low pH might account, at least in part, for the observation that patients taking PPIs have a greater likelihood of having high concentrations of trypsin in their gastric fluid. In general, the concentrations of all analytes evaluated varied over several orders of magnitude, covering a minimum of a 2000‐fold range (gastricsin) and a maximum of a 1 × 10^6^ –fold range (trypsin). Furthermore, the concentrations of various analytes were poorly correlated with one another in the samples. For example, trypsin and bile concentrations showed a significant (*P *< 0.0001) but not strong correlation (*r* = 0.54). Finally, direct assessment of bacterial concentrations by flow cytometry revealed that PPIs did not cause a profound increase in microbial load in the gastric fluid. These results further delineate the profound effects that PPI usage has on the physiology of the stomach.

## Abstract (precis)

Further delineating the profound effects that PPI usage has on the physiology of the stomach, gastric fluid was collected from patients undergoing cardiac surgery who were not (*n* = 40) or were (*n* = 25) actively taking PPIs. Proteomic analyses by mass spectrometry suggested that degradation of trypsin at low pH might account, at least in part, for the observation that patients taking PPIs have a greater likelihood of having high concentrations of trypsin in their gastric fluid. Direct assessment of bacterial concentrations by flow cytometry revealed that PPIs did not cause a profound increase in microbial load in the gastric fluid, contrary with results previously obtained using culture‐dependent methods.

## Introduction

Gastro‐esophageal reflux disease (GERD) is a diagnosis given to any person who experiences a clinically significant impairment in their well‐being due to symptoms of reflux of gastric contents (Glise and Wiklund 1997). The agreed upon mechanism whereby reflux symptoms are produced is through contact of the esophageal mucosa with acid and other contents in gastric fluid (Iwakiri et al. 2004). The prevalence of reflux symptoms in one Western population has been measured at 40%, and over one‐third of this group will experience esophagitis (Ronkainen et al. 2005). Treatment with proton pump inhibitors (PPIs) such as omeprazole is the current gold standard of drug therapy (Schindlbeck et al. 1995). PPIs are also the treatment of choice for GERD, as they are significantly more effective than H2‐receptor antagonists (Gough et al. 1996).

PPIs act to inhibit the gastric H^+^, K^+^‐ATPase (proton pump) by irreversibly inactivating the pump molecule, and are thus the most potent suppressors of gastric acid secretion (Wallace and Sharkey 2011). While PPIs are approved for the treatment of GERD, duodenal and peptic ulcers, and esophagitis, 30–40% of the prescriptions for PPIs are for off‐label uses, such as to treat gastritis (Schroder‐Bernhardi et al. 2004). One reason for this may be the accepted opinion among prescribers that PPIs have minimal side effects and are generally safe for long‐term treatment (Vanderhoff and Tahboub 2002). However, a wide range of evidence that points to consequences of long‐term PPI usage has recently gained prominence. It follows that a drug that reduces acid secretion could possibly alter a variety of metabolic processes, and recent studies demonstrate an increase in vitamin B12 and calcium deficiencies and an increased potential risk of iron deficiency in patients, particularly the elderly, following long‐term PPI use (Marcuard et al. 1994; Benito and Miller 1998; O'Connell et al. 2005; Hirschowitz et al. 2008; Fass and Sifrim 2009). PPIs have also been linked to increased chances of *Clostridium difficile* infection (Dial et al. 2004), community‐acquired pneumonia (Laheij et al. 2004), and rebound acid hypersecretion (McColl 2004), a phenomenon whereby acid secretion is above the baseline for the patient after stopping the PPI.

Despite the very widespread usage of PPIs, a broad analysis of the effects of PPIs on digestive molecules has received little attention, with most studies focused on one or two analytes. In order to provide a more detailed characterization of the effects of PPIs on gastric physiology, concentrations of common molecules in human gastric fluid, specifically pepsin, gastricsin, trypsin, and bile, were examined in patients that either did not (*n* = 40) or did (*n* = 25) take PPIs. Furthermore, the microbial growth in the samples was assessed using a direct detection method by flow cytometry. This “bottom up”, or discovery‐based approach is particularly useful in situations where profound alterations to a system (e.g., dramatic changes in pH) may substantially alter homeostasis in unexpected or difficult to predict ways, and serves as an excellent starting point for further hypothesis‐driven research.

## Materials and Methods

### Human gastric fluid samples

Human gastric fluid was collected from anonymous patients immediately prior to undergoing thoracic surgery at Duke University Medical Center. Collection of the gastric fluid was performed as a routine part of the standard preoperative procedure, and that practice was not altered for purposes of collecting the gastric fluid. Samples were collected by laboratory personnel immediately after removal from the patient's stomach (just before surgery, after anesthesia was induced). Samples were stored from 12 to 32 min at room temperature (allowing time to collect more than one sample, to transport samples back to the laboratory, assess the pH, and aliquot the sample or samples) before the samples were flash frozen with liquid nitrogen. Patients who had been on antibiotics prior to the perioperative period were excluded, and any prescriptions for acid‐blockade (e.g. proton pump inhibitors) were noted. The total number of samples collected was 65, with 40 from patients not taking proton pump inhibitors (PPIs), and 25 from patients taking PPIs. The samples were stored at −80°C until analysis. Analyses were conducted on a fraction of the samples, taking into account the fact that some of the samples were too viscous for some of the assays, some of the samples had limited volumes which prevented assessment in all assays, and results from all of the samples were not needed in order to establish statistical significance for all of the assays. The collection and analyses of these human samples was declared by the Duke Institutional Review Board to be research not involving human subjects.

### Assessment of trypsin concentrations in gastric fluid samples by ELISA

The concentration of trypsin in 63 human gastric fluid samples (24 from patients not on PPIs, and 39 from patients on PPIs) was quantified using a DuoSet ELISA Development Kit for human trypsin (R&D Systems, Minneapolis, MN). The ELISA assay was completed according to manufacturer's protocols, using the reagents provided, which included sheep anti‐human trypsin as the capture antibody, biotinylated sheep anti‐human trypsin as the detection antibody, and tetramethylbenzidine mixed with stabilized hydrogen peroxide as the substrate solution. The assay detects antigen only, and may detect trypsin which is not active, including trypsin fragments.

### Assessment of bile concentrations in gastric fluid samples

The bile concentration in 59 human gastric fluid samples (36 samples from patients not on PPIs, and 23 samples from patients on PPIs) was analyzed by an enzymatic colorimetric method using the Total Bile Acids Assay Kit (BioQuant; San Diego, CA). The assay was run on an automated platform, Cobas Integra 400 plus Analyzer (Roche Diagnostics, Indianapolis, IN), according to manufacturer's protocols but optimized for the use in BAL specimens. The lowest level of quantitation for this assay is established at 0.42 *μ*mol/L with SD of 0.01 (inter‐ and intra‐assay cv are 1.59 and 0.00%). The upper level of quantitation has been established at 9.46 *μ*mol/L (inter‐ and intra‐assay cv are 1.90 and 2.20%). The overall inter‐ and intra‐assay CVs for this test based on mid QC are as follows: intra‐assay 1.22% and interassay 2.17%. Results are calculated based on linear curve with *r*^2^ of 0.999.

### Evaluation of the particulate matter in gastric fluid by flow cytometry

The particulate matter in 29 human gastric fluid samples (19 samples from patients not on PPIs, and 10 samples from patients on PPIs) was quantified by flow cytometry. For this analysis, previously frozen aliquots of human gastric fluid were thawed in a 37°C water bath. Thirty microliters of each sample were filtered through a 35 *μ*m strainer (BD Biosciences, San Jose, CA) with three 1 mL washes of phosphate buffered saline containing 10 mg/mL bovine serum albumin and 0.2 g/L NaN_3_ to avoid clogging of the 70 mm nozzle on the flow cytometer (final sample dilution was 1:100).

The “Bacteria Counting Kit” (Invitrogen Corp; Carlsbad, CA) was utilized to evaluate the number of bacteria present. Diluted samples were divided three ways and assessed by flow cytometry as either unstained, stained, or spiked with the provided counting beads and then stained. Filtered buffer only (background), buffer with counting beads only, and cultured bacteria were used as controls. For staining, 0.5 *μ*L SYTO BC, with or without 2.5 *μ*L counting beads, were added per 500 *μ*L sample. The suspension was allowed to incubate for 5 min or longer in the dark before assessment by flow cytometry.

Flow Cytometry was performed in the Duke University Center for AIDS Research (CFAR) Flow Cytometry Core Facility on a BD FACSAriaII (BD Biosciences, San Jose, CA), and all parameters were collected on a log scale with a time duration of 60 sec. Calibration, or “quality control” particles of 0.3 *μ*m, 0.5 *μ*m, 0.8 *μ*m, and 1.0 *μ*m in diameter were a generous gift of Jeff Ware and Larry Duckett at BD Biosciences. Data were assessed using FlowJo 8.6.3 (FlowJo, LLC, Ashland, OR).

### Assessment of pepsin and gastricsin concentrations by Western blotting

The concentration of pepsin (pepsin A) in 42 human gastric fluid samples (18 samples from patients not on PPIs, and 24 samples from patients on PPIs) was assessed using a semiquantitative blotting technique. The concentration of gastricsin (pepsin C) in 36 human gastric fluid samples (16 samples from patients not on PPIs, and 20 samples from patients on PPIs) was assessed using the same approach. Thawed samples of gastric fluid were combined with Laemmli Sample Buffer (Bio‐Rad Life Science; Hercules, CA) containing 10 mmol/L dithiothreitol. The samples were boiled for 5 min, and 25 *μ*L of sample was loaded into each well of a 4 to 20% acrylamide gradient gel (Pierce Precise Protein Gels, Thermo Scientific; Rockford, IL) separated electrophoretically, and transferred to a PVDF membrane (iBlot Gel Transfer Stacks, Invitrogen Corp.). Molecular weight standards (Thermo Scientific) were run in the standard well of the preparation gel. Membranes were blocked at room temperature for 1 h using three changes of blocking buffer (StartingBlock T20 (TBS) Blocking Buffer, Thermo Scientific).

Blots were incubated for 1 h at room temperature with a mixture of antibodies specific for either pepsin (antibodies F‐16 and Q‐17; Santa Cruz Biotechnology; Santa Cruz, CA) or gastricsin (antibodies C‐17 and T‐12; Santa Cruz Biotechnology) diluted in Tris buffered saline with 0.05% Tween 20 (TBST). Blots were washed three times with TBST and incubated for 1 h at room temperature in mouse anti‐goat Immunoglobulin (Ig)G, peroxidase conjugated, (Thermo Scientific) diluted in blocking buffer. Blots were washed a total of three times with TBST over 16 h at 4°C, then washed an additional three times with TBST at room temperature over 45 min, and then three times with TBS at room temperature over 45 min. Blots were developed using SuperSignal West Pico Chemiluminescent Substrate (Thermo Scientific) for 5 min at room temperature. The blots were then exposed to CL‐XPosure Film (Thermo Scientific), and the film was developed in a Konica Processor SRX‐101A (Konica Minolta Medical Systems, Tokyo, Japan). Pepsin and gastricsin concentrations were converted to absolute units using the mass spectrometry procedures below. Quantification proved difficult as it was demonstrated that part of the insoluble material in the samples caused an artifact to appear on gel electrophoresis, and artificially lower band intensities may have occurred in some or all sample blots.

### Proteomic analysis by mass spectrometry

The proteomes of a pool of samples taken from patients not on PPIs (*n* = 11 individual samples in the pool) and of a pool of samples taken from patients on PPIs (*n* = 5 individual samples in the pool) were evaluated using mass spectrometry by the Duke University School of Medicine Proteomics Core Facility. All samples used for the study met the following criteria: (1) sufficient sample was available for analyses at the time the experiment was initiated; (2) samples from patients not on PPIs had a pH less than 4.0; and (c) samples from patients on PPIs had a pH greater than 4.0. The samples were centrifuged at 15,000 rpm, protein concentrations were measured by Bradford protein assay (Bio‐Rad Life Science), and then a set amount (10 *μ*g from each for the PPI pool and 5 *μ*g from each for the no PPI pool) was combined to make two pools. The two pools were separated into high and low molecular weight fractions using Millipore Amicon Ultra 10 kDa centrifugal filter units (EMD Millipore, Cork, Ireland) and the higher molecular weight fraction was buffer exchanged >100× into 50 mmol/L ammonium bicarbonate. A concentration was determined again by Bradford, and the entire >10 kDa fraction from each pool was then taken up in 0.1% final RapiGest (Waters Corp., Milford, MA)) to solubilize proteins, reduced in 10 mmol/L dithiothreitol, alkylated in 20 mmol/L iodoacetamide, and digested with sequencing grade modified trypsin at a 1:50 ratio of trypsin to total protein (Policy DIfGS 2014). After digestion overnight, samples were acidified to 1% trifluoroacetic acid/2% acetonitrile and heated for 2 h at 60°C to hydrolyze Rapigest, and spiked with 50 fmol Mass PREP ADH digestion standard (Waters Corp.) per microgram of total protein. Samples were then taken through Millipore C18 ZipTips for cleanup, eluting with 20% acetonitrile/0.1% trifluoroacetic acid first followed by 50% acetonitrile/0.1% trifluoroacetic acid. Eluents were dried, and the samples were reconstituted in 1% trifluoroacetic acid/2% acetonitrile.

### LC‐MS operation

Each sample was analyzed by injecting approximately 0.5 *μ*g of total digested protein onto a 75 *μ*m × 250 mm BEH C18 column (Waters Corp.) and separated using a gradient of 5–40% acetonitrile with 0.1% formic acid, with a flow rate of 0.3 *μ*L/min, in 90 min on a nanoAcquity liquid chromatograph (Waters Corp.). Electrospray ionization was used to introduce the sample in real‐time to a Q‐Tof Synapt G2 mass spectrometer (Waters Corp.). Quantitative data collection on the Synapt G2 mass spectrometer was performed in data‐independent acquisition (MS^E^) mode, using 0.9 sec alternating cycle time between low (6V) and high (27–50 V) collision energy (CE). Scans performed at low CE measure peptide accurate mass and intensity (abundance), while scans at elevated CE allow for qualitative identification of the resulting peptide fragments via database searching. Each sample was also collected in singlicate in DDA (data‐dependent acquisition) mode and in singlicate in HDMS^E^ mode with ion mobility, to generate data files for supplementary identifications.

### Quantitation of standard sample by mass spectrometry

A sample from a patient not on PPIs was used as a standard for determination of the absolute values of the relative values of pepsin and gastricsin concentrations obtained by Western blotting. For this purpose, the standard was evaluated using mass spectrometry. Upon removal from the freezer, the standard was immediately mixed with 50 mmol/L ammonium bicarbonate with protease inhibitors. The sample was centrifuged at 15,000 rpm, and the buffer was exchanged (more than 100×) into 50 mmol/L ammonium bicarbonate with protease inhibitors (Policy DIfGS 2014). Protein concentration was measured by Bradford assay. Thirty *μ*g of the sample was removed and 50 fmol of 7 undigested stable isotope‐labeled peptides matching target peptides (JPT Peptide Technologies, Berlin, Germany) was spiked in per microgram of protein. The sample was then taken to 0.1% final RapiGest to solubilize proteins, reduced, alkylated, and digested with sequencing grade modified trypsin as described above (Policy DIfGS 2014). After digestion overnight, samples were acidified to 1% trifluoroacetic acid/2% acetonitrile and heated for 2 h at 60°C to hydrolyze Rapigest. Sample was then centrifuged at 15,000 rpm for 5 min and the supernatant was pipetted into a Total Recovery LC vial (Waters Corp.).

### LC‐MS operation and standard quantitation

The standard sample was analyzed by injecting approximately 1 *μ*g of total digested protein onto a 150 *μ*m × 50 mm BEH C18 iKey device (Waters Corp.) and separated using a gradient of 5 to 40% acetonitrile with 0.1% formic acid, with a flow rate of 3 *μ*L/min, in 8.75 min and a 16 min total run time. Electrospray ionization was used to introduce the sample in real‐time to a Xevo triple quadrupole mass spectrometer (Waters Corp.). The selected reaction monitoring (SRM) method targeted seven peptides in total – one peptide to PEPA, one peptide to PEPC, three peptides to TRY1, and two peptides to TRY2. The injection was performed in triplicate to get an average response. Since pepsin and gastricsin levels were high in this particular sample, the sample was diluted 10‐fold and reanalyzed in triplicate to get a reliable quantitative value. Additional digested SIL peptides to the seven targeted peptides was spiked in to maintain the 50 fmol SIL peptides per microgram of protein concentration. The 10× diluted sample was also injected in triplicate and an average peak area was determined by loading all data using Skyline (MacLean et al. 2010), integrating light and heavy versions of each peptide. A fmol/*μ*g protein amount was estimated using a single concentration (50 fmol) of stable isotope‐labeled peptide spiked into each sample. Results of the measurement of the four digestive enzymes in the gastric fluid standard are shown in Table 1.

**Table 1.** Quantification of digestive enzymes in a gastric fluid (GF) “standard” using mass spectroscopy

	Peptide used	Peak area ratio	SD area ratio	Native (fmol)	SD (fmol)	*μ*g GF Total protein used	fmol/*μ*g	MW of protein (kDa)	ng/*μ*g	Conc (*μ*g/mL)	SD (*μ*g/mL)	% CV
Pepsin	QYFTVFDR	4.38	0.28	219.0	13.8	0.1	2190	41.98	91.9	13.8	0.87	6
Gastricsin	SYYSVYDDGNNR	19.27	3.35	963.4	167.4	0.1	9634	42.44	408.9	61.3	10.66	17
Trypsin 1	TLNNDIMLIK	1.226	0.526	61.3	26.3	1	61	26.56	1.6	0.24	0.10	43
Trypsin 2	TLDNDILLIK	0.105	0.048	5.2	2.4	1	5	26.49	0.14	0.02	0.01	45

Four enzymes in a gastric fluid (GF) “standard” were quantified using a single‐point calibration based on the constant amount of the heavy‐labeled version of each peptide that was spiked into the sample (50 fmol). The fmols of pepsin and gastricsin are from a 0.1 *μ*g total protein loading and the fmols of the trypsins are from a 1 *μ*g total protein loading on column. Concentration calculations from ng/*μ*g (peptide/total protein) to μg/mL were carried out using the 150 *μ*g total protein yield per 1 mL of delivered gastric fluid. Fifty fmols of stable isotope‐labeled peptide were used for analysis of each peptide.

### Viscometry

The relative viscosity (*η*_r_) of 21 human gastric fluid samples (10 samples from patients not on PPIs and 11 samples from patients on PPIs was determined using a falling ball viscometer. For this purpose, 2 mL glass pipets (VWR, Radnor, PA) with the tapered ends removed were used as the cylinders, and 3/32 inch diameter chrome steel ball bearings (VXB Bearing, Anaheim, CA) were utilized as the falling balls. The devices were calibrated using a series of sucrose solutions (0–50% w/v), yielding a linear standard curve of relative viscosity versus time (*r*^2^ = 0.996). Gastric fluid samples were thawed at room temperature and loaded into the glass pipettes using a syringe. Each measurement, for both standards and gastric fluid samples, was repeated 30 times, and the average time was used to construct the standard curve and to calculate the relative viscosity of each gastric fluid sample based on the standard curve.

### Statistics

The D'Agostino & Pearson omnibus normality test was utilized to assess departure from normality of all data, using an alpha of 0.05 (D'Agostino et al. 1990). The distribution of all parameters measured, with the exception of pH and viscosity, was logarithmically normal, a common phenomenon in biological data (Gronholm and Annila 2007). The log‐normal data were converted into log base 10 prior to analysis by linear regression, *t*‐tests and *F*‐tests. A linear regression model was fit to determine correlations between the data sets. An unpaired, two‐tailed *t*‐test was utilized for post hoc comparisons to assess differences in means, and the means ± standard errors are reported. An *F*‐test was utilized for assessment of differences in variances, with a *P*‐value of 0.05 taken to be significant. Welch's correction was applied to unpaired, two‐tailed t‐tests of data sets with significantly different variances. GraphPad Prism Version 5.01 (GraphPad Software, Inc., San Diego, CA) was utilized for all statistical calculations. In some cases, linear data (not log base 10) was assessed using the contingency table calculator from The College of Saint Benedict and Saint John's University, with a *P*‐value of 0.05 taken to be significant.

## Results

### Concentrations of pepsin and gastricsin in human gastric fluid by immunoblot assay

**Figure 1.**
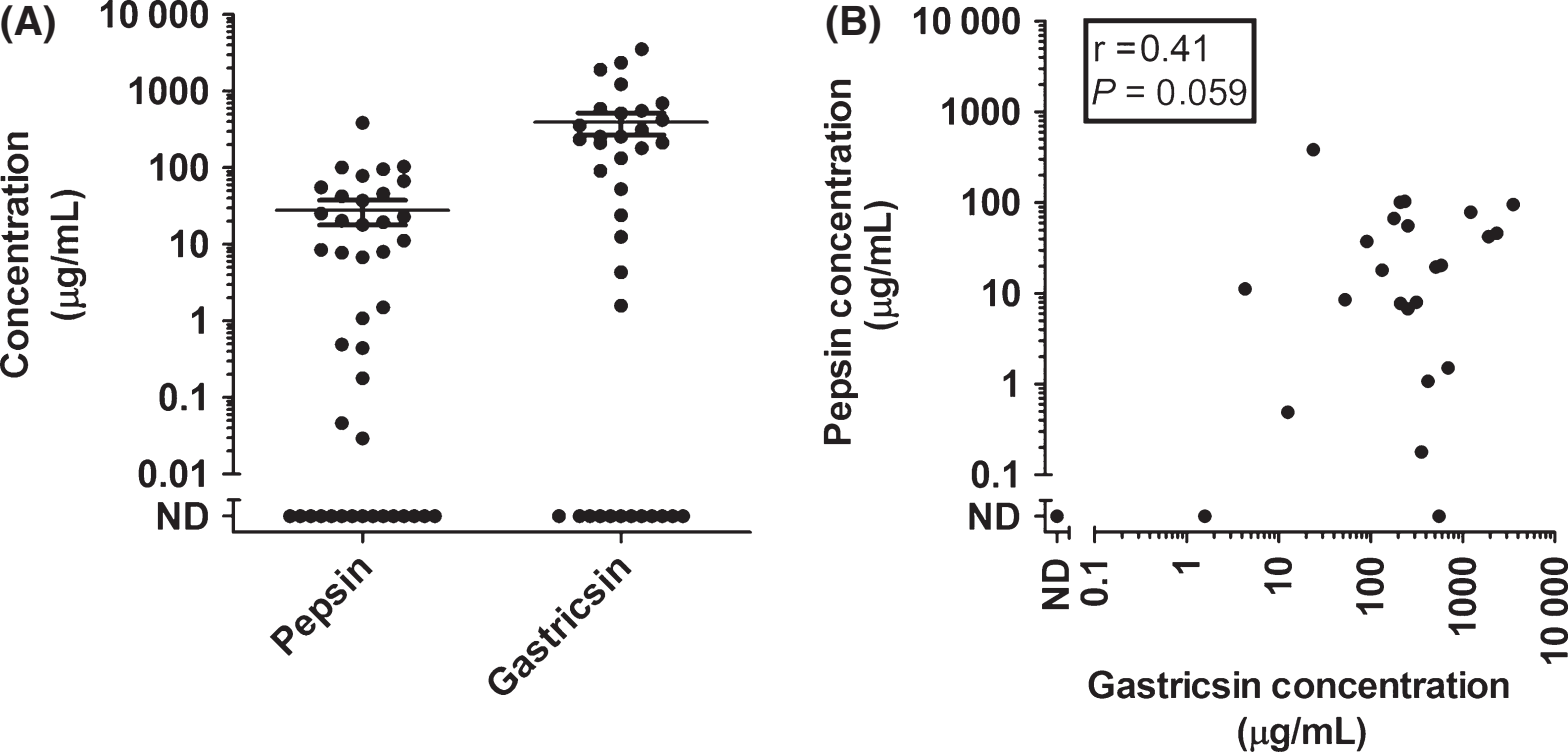
(A) Concentrations of pepsin (*n* = 42) and gastricsin (*n* = 36) in human gastric fluid. Concentrations are expressed in arbitrary units and were determined as described in the Methods. The data are normalized such that the mean concentration of each enzyme is 1.0. (ND = Not Detectable) (B) Concentration of Pepsin graphed against concentration of gastricsin in gastric fluid (*n* = 35). The single coordinate (ND, ND) corresponds to 11 data points. Samples with one or more concentrations that were not detected are not included in the regression statistics. The means and standard errors are indicated by the bars.

As shown in Fig. 1A, pepsin and gastricsin were detected in the majority of the gastric fluid samples analyzed. Pepsin was detected in 64% of the samples, and gastricsin in 67% of the samples, the enzymes being undetectable in the remaining samples. Given the very short length of time the samples were stored after removal from the patient's stomach and prior to freezing (12–32 min, see Methods), it seems likely that the failure to detect these enzymes was due to intrinsically low levels of enzymes (below the range of detection of the assay) rather than degradation of the enzymes during prolonged storage. In those samples for which both pepsin and gastricsin were detected, there was a weak, positive correlation (*r *= 0.41) between pepsin and gastricsin that was not statistically significant (*P* = 0.059) (Fig. 1B). Although concentrations of these enzymes are not significantly correlated, the association between the detection of one enzyme and the detection of the other enzyme by the blot assay is extremely significant; A post hoc contingency table constructed using detection or nondetection of pepsin versus detection or nondetection of gastricsin yielded a chi‐squared value of 27.1 (*P *< 0.001).

### Concentrations of trypsin and bile in human gastric fluid

**Figure 2.**
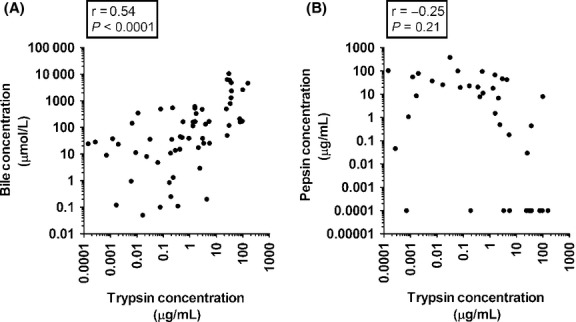
(A) Bile concentration graphed against trypsin concentration (*n* = 64). Bile concentration is expressed in micromoles per liter and was determined by an enzymatic method as described in the Methods. Trypsin concentration is expressed in micrograms per liter and was determined by ELISA as described in the Methods. (B) Trypsin concentrations graphed against pepsin concentrations (*n* = 41). Samples with a pepsin concentration that was not detected are not included in the regression statistics.

**Figure 3.**
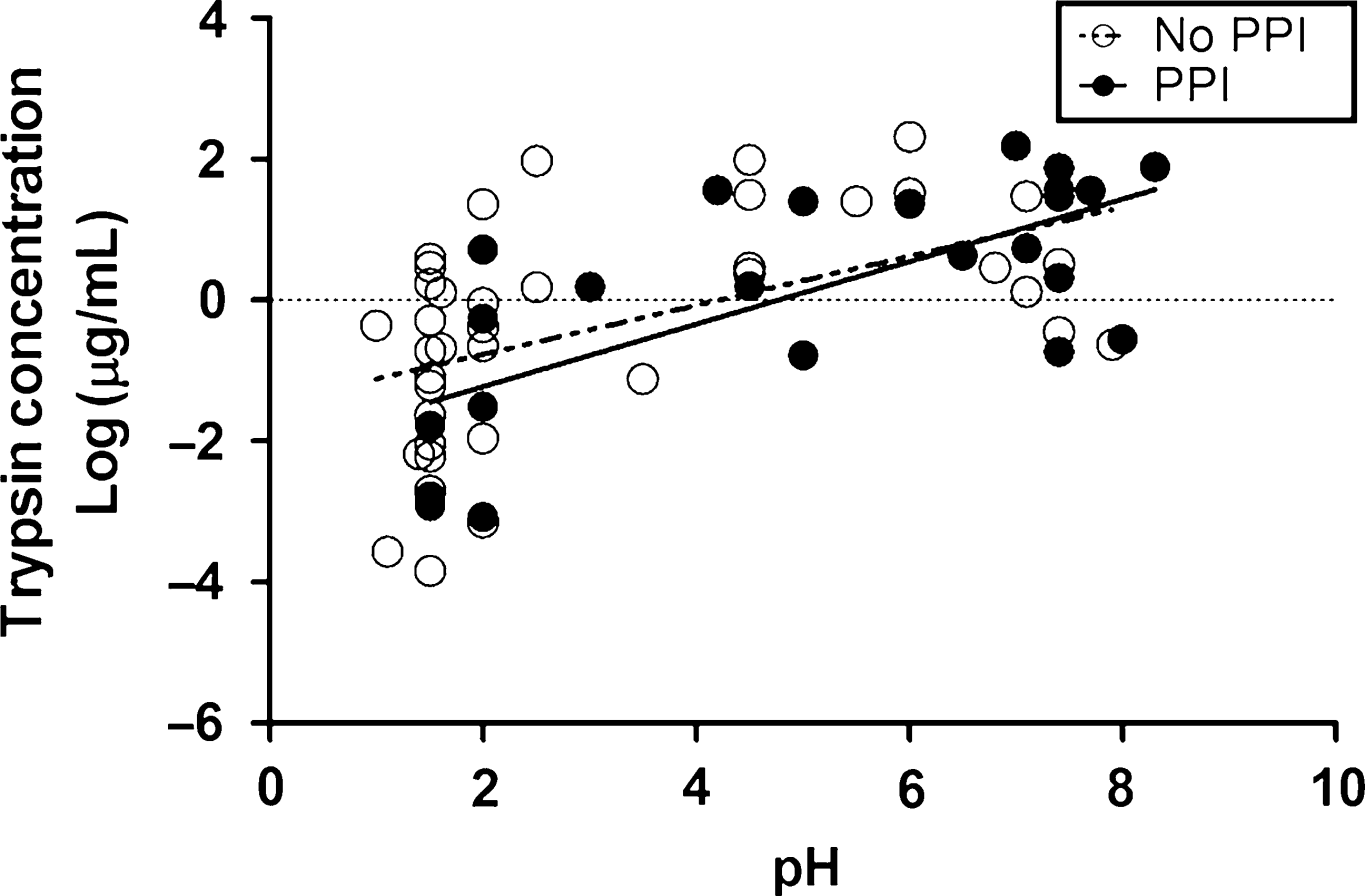
Linear regression between log Trypsin concentration versus pH. Open circles and the dashed line represent samples from patients not on PPIs (*n* = 39) and corresponding best fit linear regression line. Closed circles and the solid line represent samples from patients on PPIs (*n* = 24) and the best fit linear regression line for those data.

In addition to the gastric enzymes pepsin and gastricsin, trypsin and bile were also quantified as described in the Methods. The concentrations of trypsin and bile covered an even broader range than did the concentrations of pepsin or gastricsin. The maximum value detected for trypsin concentration was over a million‐fold more than the minimum value. The concentrations of bile detected covered a slightly smaller but still considerable range, with the maximum value being over 200,000‐fold more than the minimum value. Concentrations of trypsin and bile are significantly (*P *< 0.0001) and positively but not strongly correlated (*r* = 0.54) as shown in Fig. 2A. When the bile concentration was examined in a similar fashion, the correlation between bile concentration and pH was not statistically significant. Trypsin and pepsin concentrations were also not significantly correlated (*r* = −0.25; *P *= 0.21), as seen in Fig. 2B. However, when the two outliers with low concentrations of pepsin and low trypsin concentrations were removed, the regression became a very significant (*P *= 0.0022) with a negative correlation (*r* = −0.58). There was no statistically significant correlation between pH and the concentrations of trypsin and bile (*P *= 0.13 and *r* = 0.20; *P *= 0.28 and *r* = 0.14 respectively). To further investigate a correlation between trypsin concentration and pH, two separate regression models were fit, one with only samples from patients on PPIs and one with only samples from patients not on PPIs. When the data was split, a moderate correlation was seen in the samples from patients on PPIs (*P *= 0.0002, *r* = 0.69), and a weak correlation was seen in the samples from patients not on PPIs (*P *= 0.0011, *r* = 0.50; Fig. 3). Table 2 shows regression statistics from all of the previously mentioned linear models.

**Table 2.** Correlation between variables in gastric fluid

	Pepsin	Gastricsin	Bile	Trypsin
Pepsin		*r *= 0.41 *P* = 0.059	*r *= −0.18 *P* = 0.37	*r* = −0.25 *P* = 0.21
Gastricsin			*r* = −0.029 *P* = 0.90	*r* = −0.032 *P* = 0.88
Bile				*r *= 0.54 *P* < 0.0001
pH	*r* = −0.25 *P* = 0.21	*r* = 0.09 *P* = 0.67	*r* = 0.14 *P* = 0.28	*r *= 0.20 *P* = 0.13

A *P*‐value < 0.05 was considered significant.

### Association between PPI usage and viscosity, pH, and bacterial counts

**Figure 4.**
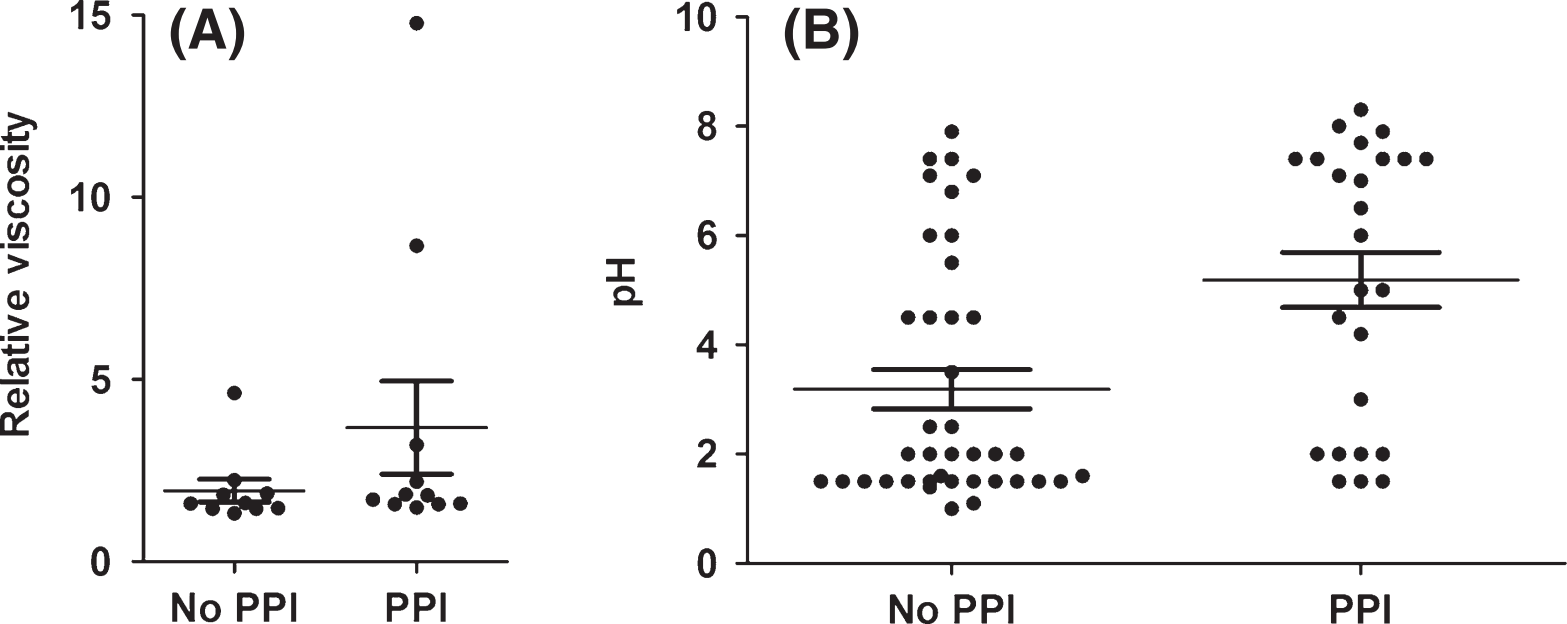
(A) pH of gastric fluid versus PPI usage (No PPI,* n* = 10; PPI,* n* = 11). (B) Relative viscosity of gastric fluid versus PPI usage (No PPI,* n* = 39; PPI,* n* = 25). Relative viscosity was calculated using a sucrose standard curve. The means and standard errors are indicated by the bars.

Most samples had a relative viscosity between 1.0 and 2.5, although a few samples were observed to have a much higher viscosity, with *η*_r_ > 4.0. Furthermore, 12.5% (three of the 24) samples were too vicious to test. The correlation between viscosity of human gastric fluid and PPI usage could not be assessed using a t‐test, due to significantly non‐normal distributions (*P *= 0.0003, D'Agostino's test for normal distribution). However, as seen in Fig. 4A, the data can be treated as bimodal using a relative viscosity of 3.0 to determine high or low viscosity. The result of a contingency table with this data was not significant (*P *= 0.16).

**Figure 5.**
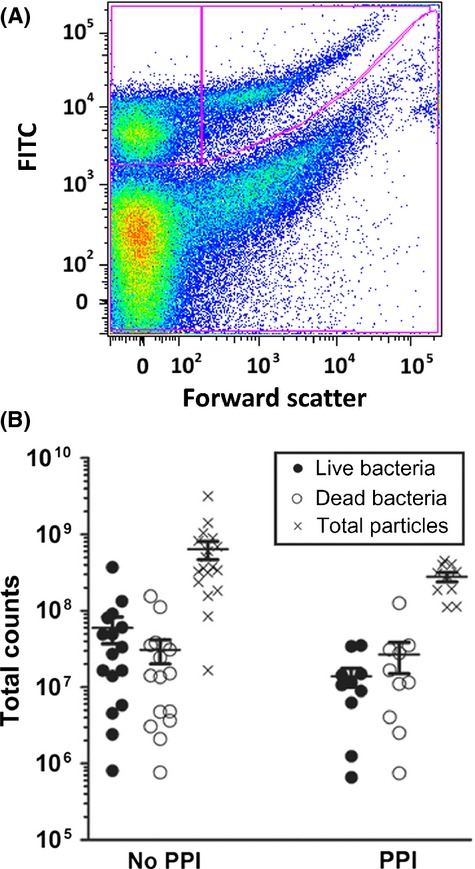
(A) Representative flow cytometry data for a gastric fluid sample. Data were collected as described above and size gates were used to separate live bacteria (top right section indicated by the lines) from bacterial cell fragments (top left section indicated by the lines). (B) Counts of live and dead bacteria versus PPI usage (No PPI,* n* = 16; PPI,* n* = 10). The count for a given parameter on each sample is represented by a circle on the graph, closed circle representing live bacteria and open circle representing dead bacteria (particles). Total particle count is represented by an X. The means and standard errors are indicated by the bars.

About two‐thirds (68%) of patients on PPIs had a high (>4.0) pH. This observation is consistent with studies showing that PPIs are not effective in 30–35% of patients (Zentilin et al. 2005; Fass and Sifrim 2009; Boeckxstaens et al. 2011), although the possibility that some patients had not actually taken their medication cannot be ruled out. In addition, about two‐thirds (67%) of patients not on PPIs had a low (<4.0) pH. The correlation between pH of human gastric fluid and PPI usage could not be assessed using a *t*‐test, due to significantly non‐normal distributions, as seen in Fig. 4B (*P *= 0.0015, D'Agostino's test for normal distribution). Treating the data as bimodal and analysis of the data with a post hoc contingency table using a cutoff of pH 4.0 for high versus low pH yielded a highly significant result (*P *= 0.007). No statistically significant differences between samples from patients not on PPIs and patients on PPIs were observed in terms of live bacterial counts, dead bacterial counts, or total particle counts (*P *= 0.43, *P *= 0.17, and *P *= 0.15 respectively; Fig. 5).

### Association between PPI usage and concentrations of various gastric fluid components

**Figure 6.**
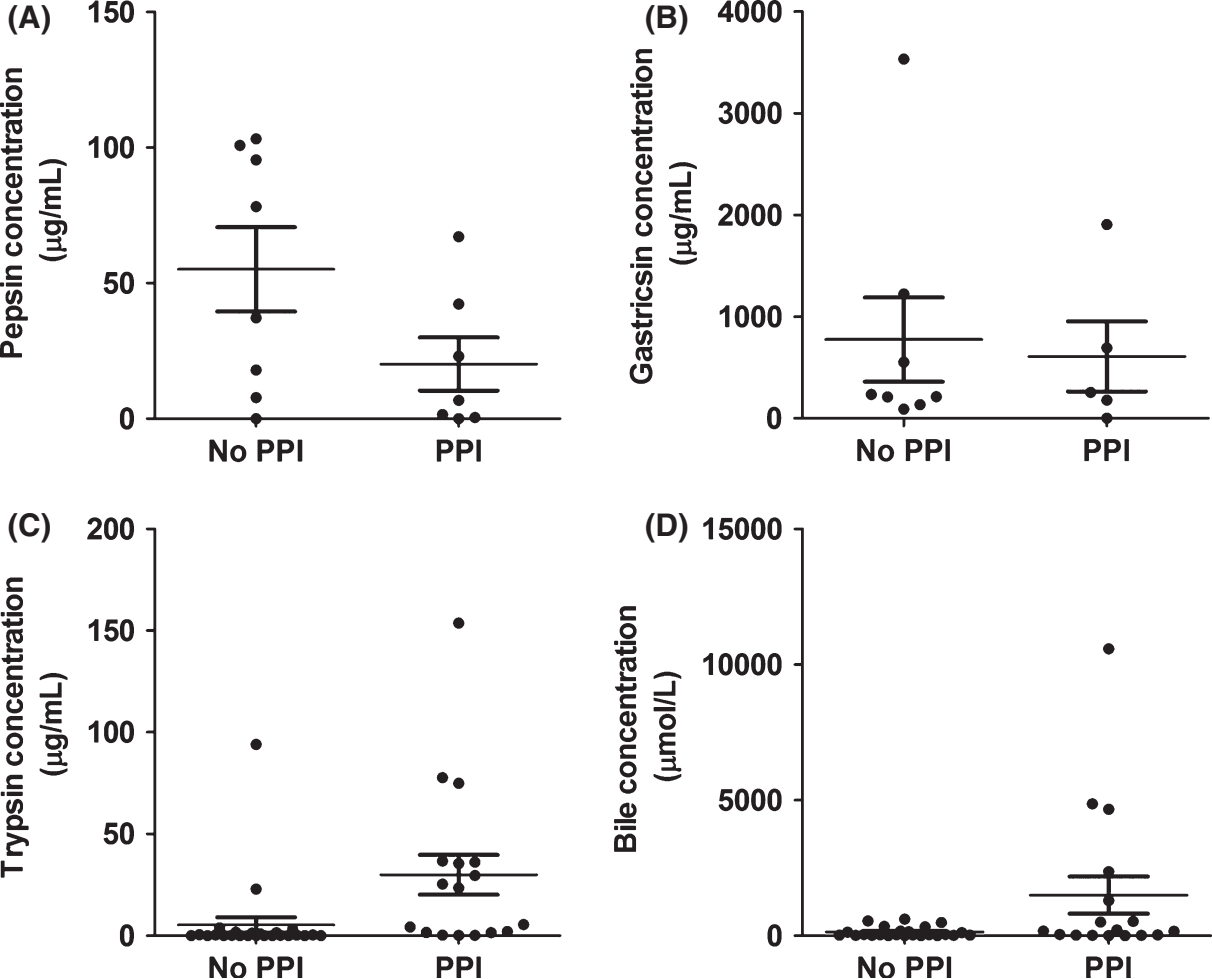
(A) Pepsin concentration in gastric fluid as a function of PPI usage (No PPI,* n* = 8; PPI,* n* = 7). (B) Gastricsin concentration in gastric fluid as a function of PPI usage (No PPI,* n* = 8; PPI,* n* = 5). (C) Trypsin concentration in gastric fluid as a function of PPI usage (No PPI,* n* = 25; PPI,* n* = 17). (D) Bile concentration in gastric fluid as a function of PPI usage (No PPI,* n* = 24; PPI,* n* = 17). Samples from patients on PPIs with a pH less than 3.0 and samples from patients not taking PPIs with a pH greater than 3.0 was excluded from the analysis (*n* = 21 samples excluded). The means and standard errors are indicated by the bars.

The association between PPI usage and the concentrations of various gastric fluid markers was assessed (Table 3). As shown in Fig. 6A, a trend toward a higher concentration of pepsin in samples from patients not taking PPIs was observed, although the difference was not statistically significant (*P *= 0.089). The gastricsin concentration showed no statistically significant association (*P *= 0.54) with PPI usage (Fig. 6B). However*,* as seen in Fig. 6C, the average trypsin concentration was higher in the samples from patients taking PPIs than in those from patients not on PPIs. Analysis of the data using a *t*‐test yielded a value of *P *< 0.0001. However, the data did not fit a normal distribution, with the majority of the samples having very low trypsin concentrations (<10 *μ*g/mL), and the rest of the samples having >20 *μ*g/mL of trypsin. Treating the data as bimodal and analysis with a post hoc contingency table yielded a highly significant result (*P *= 0.003), with the chances of having high concentration of trypsin being much greater in patients taking PPIs. Similarly, as seen in Fig. 6D, bile concentrations tended to be higher in the samples from patients taking PPIs, although the association was not statically significant (*P *= 0.14). However, analysis of the data with a post hoc contingency table revealed that the use of PPIs was associated with an increased chance (*P *= 0.005) of having high concentrations of bile (>700 *μ*mol/L) in the gastric fluid.

**Table 3.** Effect of pH alteration using PPIs on gastric fluid contents

	Mean ± SEM No PPI	Mean ± SEM PPI	*P*‐value (mean) *P*‐value (variance)
Pepsin concentration	55.1 ± 15.6 *N* = 8	20.2 ± 9.78 *N* = 7	0.089 0.218
Gastricsin concentration	773 ± 415 *N* = 8	607 ± 344 *N* = 5	0.546 0.065
Trypsin concentration	5.26 ± 3.81 *N* = 25	29.9 ± 9.80 *N* = 17	<0.0001 0.0561
Bile concentration	134 ± 38.3 *N* = 24	1500 ± 683 *N* = 17	0.140 0.051
Viscosity	1.22 ± 0.0641 *N* = 10	1.73 ± 0.354 *N* = 8	0.202
Relative viscosity	1.95 ± 0.310 *N* = 10	4.42 ± 1.71 *N* = 8	0.202
pH	3.19 ± 0.359 *N* = 39	5.19 ± 0.505 *N* = 25	0.007
Live bacteria	2.68×10^7^ ± 1.14×10^7^ *N* = 9	1.67×10^7^ ± 4.56×10^6^ *N* = 5	0.862 0.088
Dead bacteria	7.68×10^7^ ± 3.80×10^7^ *N* = 9	1.83×10^7^ ± 6.34×10^6^ *N* = 5	0.119 0.792
Total particles	6.91×10^8^ ± 2.72×10^8^ *N* = 11	2.54×10^8^ ± 6.04×10^7^ *N* = 5	0.165 0.227

Samples from patients on PPIs that was less than 3.0, or from patients not taking PPIs that was greater than 3.0 were excluded (21 samples excluded for all tests except pH *t*‐test). The *P*‐value for both the mean and the variance was determined using an unpaired t‐test and an *F*‐test respectively. A *P*‐value of less than 0.05 was considered significant.

### Differences in the proteome of pooled gastric fluid from patients not on PPIs and patients on PPIs

Samples from patients not on PPIs and from patients on PPIs were pooled separately, and divided into high (>~10 kDa) and low molecular weight (<~10 kDa) fractions as described in the Methods. The proteome of each pool was then analyzed by mass spectrometry. This arm of the project was undertaken in order to gather some broad information about potential differences between gastric fluid composition, and was not intended to be a quantitative evaluation of the variance between gastric fluid composition in patient populations on PPIs and not on PPIs. A principal component analysis of the results from the high‐molecular‐weight fractions revealed highly different profiles between the proteomes in the two pooled gastric fluid samples. Of particular interest were substantial differences in the concentrations of digestive enzymes between the two pools (Table 4). Most (14 of 16) pancreatic or hepatic enzymes identified were elevated in the pool from patients on PPIs, whereas all three gastric enzymes identified were down‐regulated in the pool from patients on PPIs. Trypsin, for example, was elevated more than sevenfold in the pool of gastric fluid from patients on PPIs compared to that from patients not on PPIs. Similarly, almost 30‐fold less pepsin was found in the pool of gastric fluid from patients on PPIs compared to that from patients not on PPIs (Table 4).

**Table 4.** Change in pancreatic/hepatic and gastric enzymes from the non‐PPI pool to the PPI pool as determined by mass spectrometry

Category	Protein	Fold change
Pancreatic/Hepatic Enzyme	*α*‐amylase 1	50.84
	Pancreatic *α*‐amylase	35.61
	Carboxypeptidase A1	16.23
	Pancreatic triacylglycerol lipase	12.84
	Carboxypeptidase B	11.96
	Chymotrypsin‐like elastase family member 3A	11.15
	Chymotrypsin‐like elastase family member 3B	7.70
	Chymotrypsin‐C	7.52
	Chymotrypsin‐like elastase family member 2A	7.50
	Trypsin‐2	7.30
	Carboxypeptidase A2	5.97
	Alcohol Dehydrogenase 1	5.55
	Chymotrpsinogen B2	3.19
	Kallikrein‐1	−4.92
	Colipase	−11.32
Gastric Enzyme	Gastricsin	−7.69
	Gastric triacylglycerol lipase	−18.11
	Pepsin A	−27.16

All differences were highly significant, with *P*‐values ranging from 7.1e‐10 to <1.4e‐45. These *P*‐values reflect differences between two pooled samples only, and do not take into account variance in the populations studied.

To probe the possibility that the pancreatic and hepatic enzymes were present but degraded in the samples from patients not on PPIs, the proteomes of the low molecular weight fractions from both pools were also assessed. In the low molecular weight fraction, peptide fragments corresponding to four of five pancreatic enzymes were found to be higher in the pool of samples obtained from patients not on PPIs, with some fragments increased 1000‐fold. This is consistent with the hypothesis that at least some pancreatic enzymes might be more rapidly degraded at low pH, and perhaps accounting in part for the relative increase in pancreatic enzymes in patients on PPIs.

A wide variety of proteins other than digestive enzymes were found to be present in different concentrations when comparing gastric fluid pooled from patients without and with PPIs. In particular, 11 keratin (type 1 and 2) proteins were decreased and 10 of 11 Immunoglobulin chains were increased in the high‐molecular‐weight pool of samples obtained from patients on PPIs compared to the patients not on PPIs. Mucin 6, a glycoprotein expressed in the gastric epithelium, was also decreased in the PPI pool.

Using an approach similar to that used for the analysis of pancreatic enzymes, the protein fragments corresponding to keratin and immunoglobulin were assessed in the low molecular weight (<10 kDa) fractions of pooled gastric fluid from patients not on PPIs and from patients on PPIs. Peptide fragments corresponding to two of three keratins were increased and from two of three immunoglobulins were increased in the non‐PPI pool. The results of the keratin proteins are consistent with the hypothesis that the gastric epithelium is shedding more rapidly at low pH, resulting in higher concentrations of epithelial proteins in the patients not on PPIs. On the other hand, greater levels of immunoglobulins in the gastric fluid of patients not on PPIs are rather unexpected, since immunoglobulins other than secretory IgA are not anticipated in the mucosal surfaces.

## Discussion

The concentrations of all analytes evaluated varied over several orders of magnitude, covering a minimum of a 2000‐fold range in the samples measured. For example, despite the effectiveness of the assays used for detection of pepsin and gastricsin over a broad range of enzyme concentrations (13,000‐ and 2000‐fold, respectively), the two enzymes could not be detected at all in 36 and 33% of the samples respectively. Thus, the range of concentrations of pepsin and gastricsin in the human gastric fluid samples evaluated was apparently greater than 13,000 and 2000‐fold respectively. Likewise, the concentrations of trypsin and of bile varied over a 1,000,000 and a 200,000‐fold range, respectively, in the samples evaluated. Concentrations of various peptides and protein fragments analyzed by mass spectrometry also varied over a wide range, up to 100 and 1000‐fold between sample pools (high‐weight and low‐weight fractions respectively). These data are consistent with previous reports, which show a wide range of concentrations of bile (Kauer et al. 1997), pepsin (Vanzant et al. 1936), trypsin (Wenger and Trowbridge 1971), and gastricsin (Chae et al. 2013) in the gastric fluid. However, these data do differ from a previous report of bacterial overgrowth in patients on PPIs (Theisen et al. 2000), perhaps due to the potential for traditional culture methods used in the previous report to detect bacteria from a stomach at neutral pH better than bacteria from a stomach at low pH. The method we employ in this study, using flow cytometry as a means of direct assessment, does not depend on the ability to culture a given bacterium.

Consistent with previous reports (Stein et al. 1992), PPI usage was associated with a significantly increased chance of having a relatively high concentration of bile in the gastric fluid. While 29% of the PPI samples had a bile concentration greater than 1000 *μ*mol/L, none of the non‐PPI samples had a bile concentration greater than 1000 *μ*mol/L (*P *= 0.005). Similarly, we found that the probability of having high concentrations of trypsin in the stomach was higher in patients using PPIs. While 47% of samples from patients on PPIs had a trypsin concentration greater than 25 *μ*g/mL, only 4% of samples from patients not on PPIs had a trypsin concentration greater than 25 *μ*g/mL, (*P *= 0.001). However, for a given pH, patients taking PPIs did not have higher concentrations of trypsin than the patients not on PPIs. It is possible that this observation is related to the pH of the gastric fluid, however, the logarithmically transformed trypsin and bile concentrations did not have significant correlations with pH (*P *= 0.13 and *P *= 0.28 respectively). The stronger correlation between trypsin and pH when examining the samples from patients on and off PPIs individually (*P *= 0.0002, *r* = 0.69 and *P *= 0.0011, *r* = 0.50, respectively), combined with the significantly increased chance of having relatively high trypsin concentrations when taking PPIs, suggests two possible mechanisms: more trypsin might be refluxed from the duodenum to the stomach in some patients taking PPIs, and trypsin could be more rapidly degraded in the stomach at low pH than at high pH. The mass spectrometry data supports this latter possibility of degradation at lower pH, since trypsin and other pancreatic enzymes of the high weight pool of samples from patients on PPIs were increased, and the degraded peptides corresponding to the same were increased in the pool of samples from patients not on PPIs.

The concentrations of various analytes were generally not correlated with one another in the samples evaluated (Table 2), reflecting the complex and dynamic properties of the digestive tract. For example, studies show that gallbladder emptying and consequential increases in duodenal bile concentration are stimulated by consumption of fats (Fisher et al. 1987) and that the duodenal and gastric concentrations of bile can vary a great deal from patient to patient because of this fat correlation. Even so, there was a significant (*P *< 0.0001) but not strong (*r* = 0.54) correlation between trypsin and bile concentrations, suggesting that the two analytes might share some factors which affect their presence in gastric fluid. Patients who reflux from the duodenum into the stomach probably reflux all of the duodenal contents. This would suggest a correlation between duodenogastric reflux and PPI usage; however, no causal relationship is implied. This association could be explained by one of two mechanisms. PPIs are often prescribed to patients with GERD, and a major cause of damage from GERD is a mechanically defective lower esophageal (or cardiac) sphincter (Stein et al. 1992). It may be that these patients also have a weak or defective pyloric sphincter, causing reflux up from the upper small bowel. There is also the possibility that PPIs could have a direct role in the increased concentrations of pancreatic and hepatic secretions, although this explanation seems unlikely.

Patients who take PPIs normally have regular gastroesophageal reflux, causing injury to the esophagus, but recent evidence points to a role of trypsin and bile in esophageal damage. Bile salts and trypsin have demonstrated a significant role in the pathogenesis of alkaline reflux esophagitis (Salo and Kivilaakso 1983), and trypsin has also been shown to cause mucosal damage in esophagitis (Naito et al. 2006). GERD affects the lungs in addition to the esophagus, and trypsin and bile have been shown to negatively affect the lungs as well. For example, induced aspiration in rats with pancreatic enzymes such as trypsin resulted in decreased counts of leukocytes, neutrophils, and platelets in the peripheral blood. Furthermore, lung compliance decreased and resistance increased significantly, and histological examination of the lungs showed edema, hemorrhage, and alveolar inflammation (Kiyonari et al. 2000). Bronchiolitis obliterans syndrome (BOS) has recently been connected to aspiration of bile. In multiple studies, an increase in bile levels in BAL has shown to be directly associated with the development of BOS (D'Ovidio et al. 2005; Blondeau et al. 2008). The correlation between trypsin and bile concentrations, added to the knowledge that these molecules both induce or exacerbate lung injury, provides a picture that duodenogastric reflux in combination with aspiration might be exceptionally deleterious to the lung. The increased concentrations of trypsin and bile in patients on PPIs were accompanied by a decrease in pepsin. Pepsin, in a study by Popper and colleagues, was demonstrated to accelerate lung damage and fibrosis compared to acid alone (Popper et al. 1986). If the PPIs were shown to have a direct role in increasing reflux up from the small bowel, then it is possible that PPIs could accelerate lung damage due to gastroesophageal reflux and aspiration. However, it is possible that the decreased pepsin concentrations in patients taking PPIs might offset the negative consequences of increased trypsin and bile concentrations.

It is hoped that this study will encourage other studies looking in greater detail at the physiologic consequences of PPI usage. This study has some limitations in that no information was collected regarding the demographics of the population studied (e.g., age, smoking habits, BMI), the dosage of drug, the specific PPI used, and whether the patients were compliant with their medications. Furthermore, some of the analyses involved small numbers, and the patients were not “typical” in the sense that they were patients undergoing surgery. Future studies using different and perhaps larger patient populations with more demographic information may provide additional insight into this important issue. In addition, the effect of H_2_ receptor antagonists (H2RAs) on gastric physiology was not evaluated in this study, and merits evaluation in future studies.

## Acknowledgments

The Duke University Center for AIDS Research (CFAR) Flow Cytometry Core Facility is funded in part by Grant P30 AI 64518 from the National Institutes of Health.

## Conflict of Interest

None declared.
